# Telbivudine Treatment during Late Pregnancy Prevents Mother-to-Child Transmission of Hepatitis B Virus: A Retrospective Study

**DOI:** 10.1155/2019/9046260

**Published:** 2019-07-09

**Authors:** Mengzhi Cai, Yanli Hao, Jianxin Zhong, Wei Yao, Xia Cao, Guifang Gu, Gang Qin

**Affiliations:** ^1^Center for Liver Diseases, Nantong Third People's Hospital, Nantong University, Nantong 226006, China; ^2^Department of Obstetrics and Gynaecology, Affiliated Hospital of Nantong University, Nantong 226001, China; ^3^Department of Obstetrics and Gynaecology, Nantong Third People's Hospital, Nantong University, Nantong 226006, China

## Abstract

**Purpose:**

To investigate the efficacy of telbivudine (LdT) in blocking mother-to-child transmission (MTCT) of hepatitis B virus (HBV) during late pregnancy.

**Methods:**

A total of 651 pregnant women aged 18-40 in Nantong Third People's Hospital and Hospital affiliated to Nantong University with positive hepatitis B surface antigen (HBsAg) and HBV DNA were enrolled between January 2011 and December 2015. Patients with HBV DNA≥10^6^ copies/mL (n=251) received LdT during late pregnancy according to the patients' will, while 136 high viral patients with HBV DNA≥10^6^ copies/mL who did not take LdT therapy and 268 low viral patients with HBV DNA<10^6^ copies/mL served as the controls.

**Results:**

At 7 months and 1 year postpartum, the basal HBV DNA serum level of treated patients declined significantly (*P*<0.001), while no obvious decline was observed in the untreated high viraemic controls (*P*<0.05) and untreated low viraemic controls (*P*<0.05). Only 1 infant (0.4%) in LdT group was HBsAg positive at 7 months, while 14 (5.2%) were in the untreated low viraemic controls (*P*<0.001) and 15 (11.0%) were in untreated high viraemic controls (*P*<0.001).

**Conclusion:**

For pregnant women with HBV DNA≥10^6^ copies/mL, the use of LdT during late pregnancy could effectively reduce the MTCT rate of HBV.

## 1. Introduction

Hepatitis B virus (HBV) infection has always been a threat to public health. More than 240 million people are affected by chronic HBV all over the world [1]. About 650 thousand people died from HBV-related liver cirrhosis and hepatic cellular carcinoma (HCC) every year [2]. About 2% to 10% of the patients with chronic hepatitis B (CHB) may progress to liver cirrhosis every year. For noncirrhotic patients with HBV infection, the annual incidence of HCC is 0.5% to 1%. For cirrhotic patients with HBV infection, the annual incidence of HCC is 3% to 6% [3]. The proportion of liver cirrhosis and HCC caused by HBV infection is 30% and 45% worldwide [4], while in China the proportion is 60% and 80% [5, 6]. In countries with a high prevalence of HBV, mother-to-child transmission (MTCT) still accounts for most cases with HBV infections [7, 8]. Most of perinatal infections may progress to CHB and then evolve to HBV-related liver cirrhosis and cancer [9–12]. MTCT, or perinatal transmission, or vertical transmission refers to detectable HBV DNA and/or hepatitis B surface antigen (HBsAg) in peripheral serum samples of infants at 7 months [13, 14]. The application of HBV vaccine (0, 1, and 6 months) and hepatitis B immune globulin (HBIG) for infants could decrease the risk of MTCT from 90% to 5%-10% [15–18], but MTCT still occurs in infants born to women with high HBV viral load [10, 15, 19], and the risk of MTCT is particularly high for women with HBV viral load (≥10^6^ of DNA/mL) [20]. Now, the application of antiviral drugs during late pregnancy to block MTCT is recommended in many countries, including China [9, 21]. Telbivudine (LdT) and Tenofovir disoproxil fumarate (TDF), the only category B (no risk in animal studies, but unknown in human) drugs according to the Food and Drug Administration (FDA), remain the first choice for antiviral therapy in China [22].

We conducted this retrospective cohort study based on a total of 651 pregnant women aged 18-40 with positive HBsAg and HBV DNA from Nantong Third People's Hospital and Affiliated Hospital of Nantong University between January 2011 and December 2015 to study the efficacy of LdT in blocking MTCT of HBV during late pregnancy. Clinical data of the pregnant women were all available during pregnancy and within 1 year postpartum; clinical data of the infants were available at birth and 7 months postpartum.

## 2. Methods

### 2.1. Study Design

A retrospective review of the treatment and follow-up of patients aged 18-40 with positive HBsAg and HBV DNA and their respective infants were undertaken in Nantong Third People's Hospital and Affiliated Hospital of Nantong University from January 2011 to December 2015. All participants were identified using codes from the International Classification of Disease, 10th edition (ICD-10). The codes used to identify viral hepatitis complicating pregnancy, childbirth, and the puerperium were O95.482 [23, 24]. According to the above method, 651 pregnant women satisfied the inclusion criteria and were enrolled. Among participants of this study, 247 were from Nantong Third People's Hospital and 404 were from Hospital affiliated to Nantong University.

All subjects in this study fulfilled the following criteria: (i) 18-40 years old; (ii) HBsAg positive; (iii) HBV DNA positive; (iv) clinical data of the pregnant women all available during pregnancy and 1 year postpartum and clinical data of the infants available at birth and at 7 months postpartum. The exclusion criteria were as follows: (i) antiviral treatment within 1 year; (ii) coinfection with hepatitis A, C, D, and E or human immunodeficiency virus (HIV).

The protocol of this retrospective study without any intervention was reviewed and approved by both institutional review boards (IRBs). All data were processed anonymously. Informed consent was waived by the same IRBs.

### 2.2. Data Collection

Data regarding perinatal HBV infection were collected and submitted by dedicated personnel from the two participating hospitals: All subjects were classified into three groups. 264 patients with HBV DNA<10^6^ copies/mL served as group A. 136 patients with high viral load (HBV DNA≥10^6^ copies/mL) who did not receive LdT therapy represented group B. 251 patients with high viral load (HBV DNA≥10^6^ copies/mL) who received LdT therapy were classified in group C.

Patients in group A took the prenatal examination regularly. Patients in group B were prescribed anti-inflammatory and hepatoprotective drugs and did not take antiviral drugs. Patients in group C received LdT (Novartis, Swiss) 600 mg orally daily at 28-32 weeks of gestation [24–26]. Patients with elevated ALT level received neo-minophagen C or magnesium isoglycyrrhizinate, and polyene phosphatidylcholine. All the infants received 100 IU hepatitis B immune globulin (HBIG) and 10 *μ*g of HBV vaccine intramuscularly within 12 hours after birth, and then 10 ug additional vaccines at 1 and 6 months of age [27]. 85.7% patients in group C continued to take LdT or switched to other antiviral drugs at 1 year postpartum.

The HBV serum markers (HBV-M) were detected by enzyme-linked immunosorbent assay kit (Abbott Labs, Chicago, USA). HBV DNA was detected by the real-time quantitative polymerase chain reaction (qPCR) amplification kit (detectable baseline of HBV DNA was 10^3^ copies/mL, Kehua Biological Company, Shanghai, China).

### 2.3. Efficacy Assessment

The primary efficacy measure was the MTCT rate. Secondary efficacy measure was the reduction of serum HBV DNA levels (decrease≥2 log10copies/mL from baseline), rate of undetectable HBV DNA (HBV DNA<10^3^ copies/mL), the normalization rate of ALT, and other parameters such as adverse events, gestation age, incidence of postpartum hemorrhage, rate of meconium staining of the amniotic fluid, premature rupture of membrane, and mode of delivery. Data regarding the infant assessment were Apgar score (5 minute), the birth weight, the rate of low birth weight infant, the incidence of preterm birth, and signs of fetal distress.

### 2.4. Statistical Methods

Continuous variables were summarized as mean ± standard deviation and dependent *t* test was used for comparisons of two groups. Categorical variables were summarized as number or percentage and chi-square test was used for in-between group comparisons. A* P*-value of <0.05 was considered significant. Logistic regression was used to study the potential risk factors of infants with HBV infection. All analyses were conducted with Stata Software version 13.1 (StataCrop, USA).

## 3. Results

### 3.1. Baseline Characteristics of Pregnant Women and Newborn Infants

**Figure 1:**
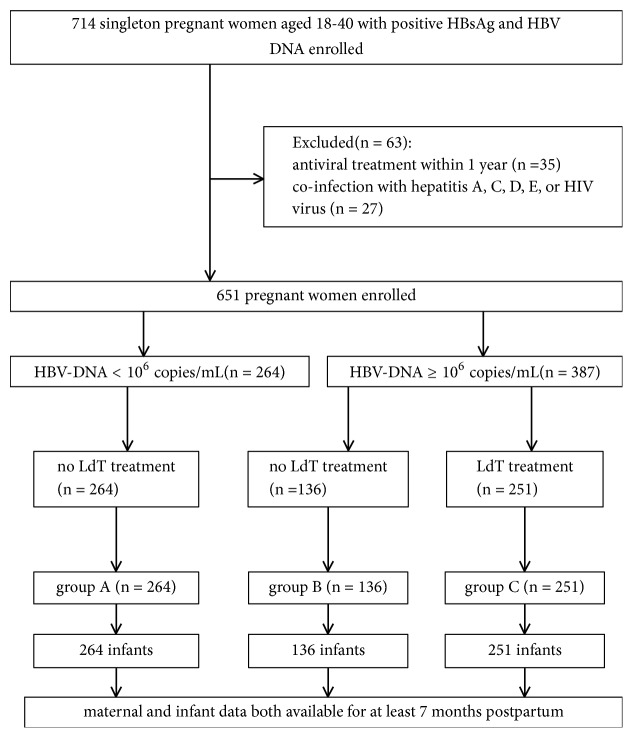
Flowchart of patients' enrollment. HBV, hepatitis B virus; HBsAg, hepatitis B surface antigen; LdT, telbivudine.

In total, 714 pregnant women were enrolled, and 651 were qualified for the final analysis. 264 low viral patients (group A) and 136 high viral patients who did not receive antiviral therapy (group B) served as controls. 251 high viral patients who received antiviral therapy at the third trimester were the treatment group (group C) (Figure 1).

There were no significant differences among the pregnant women of the three groups regarding age, primipara status, mode of delivery, premature rupture of membrane, meconium staining of the amniotic fluid (II-III degree) [28], and postpartum hemorrhage. The gestation week of the patients in group A was longer than that in group B and group C (6.7% vs. 12.5%,* P*<0.05; 12.5% vs. 12.4%,* P*>0.05). There were also no significant differences among the newborn babies Apgar scores, low birth weight, premature birth, and fetal distress (Table 1).

### 3.2. Efficacy of Telbivudine: Mothers

The HBV DNA level of the patients is shown in Table 2. There was a significant difference in the reduction of serum HBV DNA (decrease≥2 log10copies/mL from baseline). 237 (94.4%) patients in group C had a significant decline of serum HBV DNA (decrease≥2 log10 copies/mL from baseline) prior to their delivery while no patients in groups A and B had a reduction of HBV DNA (94.4% vs. 0% vs. 0%,* P*<0.001). 85.7% patients in group C continued to take LdT or switched to other antiviral drugs at 1 year postpartum. The lower detection limit of HBV DNA was 10^3^ copies/mL. In group C, and 21 (8.4%) patients had undetectable HBV DNA before delivery. The number increased to 132 (52.6%) at 7 months postpartum and 151 (60.2%) at 1 year postpartum, suggesting the effectiveness of telbivudine therapy (Table 2).

The ALT level of the patients in group A did not decline. The ALT of the patients in group B decreased after the use of liver-protection drugs. In group C, 134 (53.4%) patients achieved normal ALT level before delivery. More than 80% patients in group C kept normal level of ALT at 7 months postpartum (83.7%) and 1 year postpartum (87.3%) (Table 3).

### 3.3. Efficacy of Telbivudine: Infants

651 newborn babies were born from 651 pregnant women. Data regarding 7 months follow-up suggested that the MTCT rate in group C was 0.4% (infants with HBsAg positive at 7 months of age), compared with 14 (5.3%) and 15 (11.0%) in group A and group B mothers (11.0% vs. 0.4%,* P*<0.001) (Table 4).

Univariate analysis revealed that LdT treatment was associated with lower risk (odds ratio=0.05, 95% confidence interval 0.01-0.38;* P*<0.001) and HBV DNA levels were associated with higher risk (odds ratio=1.39, 95% confidence interval 1.17-1.64) of infant HBsAg positivity at 7 months. Multivariate analysis showed that only two factors were independently associated with MTCT of HBV: high HBV DNA before delivery (OR 1.21, 95% CI 1.02-1.44) and LdT treatment (OR 0.07, 95% CI 0.01-0.55) (Table 5).

## 4. Discussion

Maternal viral load is the most important risk factor for MTCT of HBV. Studies have shown that patients with high viral load (HBV DNA≥10^6^ copies/mL) are closely correlated with HBV infection of their infants [5, 10, 20, 29, 30]. Neonatal active-passive combination immunization cannot completely block MTCT [17, 31, 32]. NAs treatment (such as LdT and TDF) for patients with high viral load (HBV DNA≥10^6^ copies/mL) during late pregnancy can effectively block the MTCT of HBV and has been reported by several other studies [14, 33, 34]. In our study, we confirmed that maternal viral load was an independent risk factor for MTCT. Furthermore, we have shown LdT treatment could significantly reduce the MTCT incidence.

MTCT rate of HBV was 0% for pregnant women with low viral load [17, 35, 36]. On the other hand, the MTCT rate in our cohort was 5.30% (14/264) for infants born to mothers who were in the untreated-low group. A meta-analysis from Chen et al. [37] reported that the incidence of MTCT for mother with HBV DNA <10^6^ copies/mL was 2.75% (95%CI, 1.20-4.31), indicating that pregnant women with low viral load can still infect their babies with HBV. Whether antiviral treatment is needed for low viral patients requires further investigations.

Maternal HBeAg positivity was also reported as an independent risk factor for MTCT in several studies [38–40]. A study from Chen* et al*. [41] reported that MTCT rate of HBeAg positive mothers (54/583) was significantly higher than that of HBeAg negative mothers (4/1773) (9.26% vs. 0.23%,* P*<0.001). In our study, the MTCT rate was 4.73% for mothers with positive HBeAg, compared with 4.3% for mothers with negative HBeAg (4.73% vs. 4.3%).

There are limited data concerning the safety of LdT treatment during late pregnancy [20, 34, 42]. Recently, a multicenter study from Hu Y* et al*. [29] found that 2.3% (3/128) of the infants in the treatment group had severe adverse events (one case had congenital right ear loss, another one was diagnosed with cerebral palsy at 6 months, and the other one had motor dysplasia (could not stand alone)), while no obvious adverse events were observed in the control group (*P*<0.001). As a retrospective study, we could not include prenatal adverse events such as spontaneous abortion and stillbirth. However, we did not identify any congenital malformations or mental retardation among the 251 infants born from mothers who were in the LdT-treated group. Yet, studies regarding the long-term safety of antiviral therapy during late pregnancy to block MTCT of HBV still need more data and long-time follow-up in the future.

In conclusion, maternal antiviral treatment with LdT during late pregnancy can effectively reduce the viral load and promote liver function recovery. Moreover, it can also reduce the MTCT rate of HBV.

## Figures and Tables

**Table 1:** Characteristics of pregnant women and newborn infants from group A, B and C.

	Group A (n=264)	Group B (n=136)	Group C (n=251)	*P* Group A vs. B	*P* Group B vs. C
Maternal					
Primipara	193 (73.1%)	109 (80.1%)	191 (76.1%)	0.121	0.362
Age (years)	27.6±4.6	27.7±4.3	27.5±4.2	0.841	0.444
Week of gestation	39.29±1.07	38.91±1.27	38.99±1.32	0.002	0.564
Vaginal delivery	64(24.1%)	25(18.4%)	59(23.5%)	0.182	0.395
Premature rupture of membrane	18 (6.8%)	17(12.5%)	31 (12.4%)	0.057	0.966
Meconium staining of the amniotic fluid (II-III degree)	53 (20.1%)	27(19.9%)	50 (19.9%)	0.958	0.987
Postpartum hemorrhage	15 (5.7%)	8 (5.9%)	14 (5.6%)	0.935	0.902
Neonatal					
Apgar score (5 min)	9.89±0.49	9.88±0.43	9.90±0.50	0.841	0.694
Preterm birth	7 (2.7%)	7 (5.1%)	17 (6.0%)	0.198	0.527
Fetal distress	56 (21.2%)	29 (21.3%)	51 (20.3%)	0.979	0.816

Values are reported as mean ± standard deviation, compared with t test; or number (percentage), compared with chi-square test or fisher's test, unless otherwise indicated. n, number of patients.

**Table 2:** HBV DNA levels of the pregnant women during pregnancy and postpartum.

	Group A (n=264)	Group B (n=136)	Group C (n=251)	*P* Group B vs. C
baseline				
HBV DNA (log_10_ copies/mL)	3.51±0.58	7.89±0.80	7.73±0.65	0.034
before delivery				
HBV DNA (log^10^ copies/mL)	3.89±0.45	7.75±0.80	3.60±0.36	<0.001
HBV DNA decline ≥2log_10_ copies/mL from baseline (n, %)	0	0	237 (94.4%)	<0.001
Undetectable HBV DNA	60 (22.7%)	0	21 (8.4%)	
7 months postpartum				
HBV DNA (log_10_ copies/mL)	3.85±0.47	7.74±0.89	3.25±0.21	<0.001
HBV DNA decline ≥2 log_10_ copies/mL from baseline (n, %)	0	0	251 (100%)	<0.001
Undetectable HBV DNA	63 (23.9%)	0	132 (52.6%)	<0.001
1 year postpartum				
HBV DNA (log_10_ copies/mL)	3.83±0.47	7.71±0.80	3.21±0.20	<0.001
HBV DNA decline ≥ 2 log_10_ copies/mL from baseline (n, %)	0	0	251 (100%)	<0.001
Undetectable HBV DNA	64 (24.2%)	0	151 (60.2%)	<0.001

Values are reported as mean ± standard deviation, compared with t test; or number (percentage), compared with chi-square test, unless otherwise indicated. Undetectable HBV DNA: HBV DNA < 10^3^ copies/mL. n, number of patients.

**Table 3:** ALT levels of the pregnant women during pregnancy and postpartum.

	Group A (n=264)	Group B (n=136)	Group C (n=251)	*P* Group B vs. C
Baseline (U/L)	33.13±9.95	127.49±68.23	120.63±67.07	0.340
0-40	218 (82.6%)	0	0	0.369
41-80	46 (17.4%)	42 (30.9%)	90 (35.9%)	
81-200	0	73 (53.68%)	134 (53.39%)	
201-400	0	21 (15.44%)	26 (10.36%)	
401-	0	0	1 (0.40%)	
Before delivery (U/L)	32.89±9.25	53.13±14.88	42.00±12.01	<0.001
0-40	219 (83.0%)	31 (22.8%)	134 (53.4%)	<0.001
41-80	45 (17.0%)	97 (71.3%)	114 (45.4%)	
81-200	0	8 (5.9%)	3 (1.2%)	
201-400	0	0	0	
401-	0	0	0	
7 months postpartum (U/L)	27.24±8.21	60.18±14.12	31.06±1.66	<0.001
0-40	241 (91.3%)	3 (2.2%)	210 (83.7%)	<0.001
41-80	23 (8.7%)	116 (85.3%)	41 (16.3%)	
81-200	0	17(12.5%)	0	
201-400	0	0	0	
401-	0	0	0	
1 year postpartum (U/L)	26.54±7.85	58.83±1.16	29.01±8.66	<0.001
0-40	243 (92.0%)	4 (2.9%)	219 (87.3%)	<0.001
41-80	21 (8.0%)	119 (87.5%)	32 (12.7%)	
81-200	0	13(9.6%)	0	
201-400	0	0	0	
401-	0	0	0	

Values are reported as mean ± standard deviation, compared with t test; or number (percentage), compared with chi-square test or fisher's test, unless otherwise indicated. n, number of patients; ALT, alanine aminotransferase.

**Table 4:** HBsAg and anti-HBs status of the infants.

	Group A (n=264)	Group B (n=136)	Group C (n=251)	*P* Group B vs. C
HBsAg				
At birth	16 (6.06%)	17 (12.50%)	15 (5.98%)	0.026
7 months	14 (5.30%)	15 (11.03%)	1 (0.40%)	<0.001
Anti-HBs				
7 months	232 (87.88%)	131 (96.32%)	249 (99.20%)	0.055

Values are reported as number (percentage), compared with chi-square test or fisher's test. n, number of patients.

**Table 5:** Risk factors for MTCT of HBV.

	Case/exposed, %	Univariate OR (95% CI)	Adjusted OR (95% CI)
Age			
<35	27/595, 4.54%	1	
≥35	3/56, 5.36%	1.19 (0.35-4.06)	
HBeAg status			
Negative	8/186, 4.30%	1	
Positive	22/465, 4.73%	1.10 (0.46-2.92)	
HBV DNA before delivery			
<10^6^ copies/mL	14/264, 5.30%	1	
≥10^6^ copies/mL	16/387, 4.13%	1.39 (1.17-1.64)	1.21 (1.02-1.44)
ALT at baseline (U/L)			
0-40	10/218, 4.59%	1	
41-80	9/178, 5.06%	1.11 (0.44-2.79)	
>80	11/255, 4.31%	0.94 (0.39-2.25)	
Telbivudine treatment			
No	29/400, 7.25%	1	
Yes	1/251, 0.40%	0.05 (0.01-0.38)	0.07 (0.01-0.55)
Gestation week			
≥37 weeks	29/620, 4.68%	1	
<37 weeks	1/31, 3.23%	0.68 (0.09-5.16)	
Mode of delivery			
Vaginal delivery	22/148, 14.86%	1	
Caesarean section	8/503, 1.59%	0.09 (0.03-0.22)	0.72 (0.31-1.69)
Fetal distress			
No	27/515, 5.24%	1	
Yes	3/136, 2.21%	0.41 (0.12-1.36)	
Postpartum hemorrhage			
No	28/614, 4.56%	1	
Yes	2/37, 5.41%	1.20 (0.27-5.22)	
Meconium staining of the amniotic fluid			
No	27/521, 5.18%	1	
Yes	3/130, 2.31%	0.43 (0.13-1.45)	
Premature rupture of membrane			
No	28/585, 4.79%	1	
Yes	2/66, 3.03%	0.62 (0.14-2.67)	

## Data Availability

The data used to support the findings of this study are available from the corresponding author upon request.
